# THE IMPACT OF SOCIOECONOMIC STATUS ON AGE TRAJECTORY OF BODY MASS INDEX AMONG MIDDLE-AGED AND OLDER ADULTS

**DOI:** 10.1093/geroni/igz038.532

**Published:** 2019-11-08

**Authors:** Yu-Chun Lin, Yu-Hung Chang

**Affiliations:** 1 China Medical University Hospital, Taichung, Taiwan; 2 China Medical University, Taichung City, Taiwan

## Abstract

Body weight tends to decrease along with age. Weight loss and low body mass index (BMI) in the elderly, associated with socioeconomic status, are both strong predictors of subsequent mortality.This study aims to investigate the relation between income and BMI changes in later life. We used data from the Taiwan Longitudinal Study in Aging (TLSA) from 1999 to 2007. There were 5,131 participants aged 50 and over, who were excluded for those without primary study variables. Income was evaluated by asking the amount of annual income, including salary, pension, rent, interest, welfare benefit, etc. Participants’ BMI were assessed in each survey. General estimating equation models were performed to examine the association between age, annual income, and their interaction with BMI adjusting for covariates including sex, education, marital status, smoking, exercise frequency, appetite, and number of comorbidities. Totaling 11,350 person-times was in three follow-up surveys, which left 9,723 person-times of observations after exclusion. After adjusted for covariates, the low income group compared to the higher income, would have higher estimated BMI at age of 50 (BMI= 24.75 kg/m2 and 24.19 kg/m2 respectively), and more rapid reduction (-0.08 kg/m2 per year), while relatively stable BMI was found in higher income group (0.01 kg/m2 per year, slope difference= 0.10 kg/m2 per year, 95% confidence interval [CI] = 0.03-0.17). In conclusion, compared to invariable BMI observed among individuals with higher financial status, the economically disadvantaged experienced BMI decline with age among middle-aged and older adults.

